# Patient safety in distal femoral resection knee arthroplasty for non-tumor indications: a single-center consecutive cohort study of 45 patients

**DOI:** 10.1186/s12891-022-05100-7

**Published:** 2022-03-03

**Authors:** Yasemin Corap, Michael Brix, Claus Emmeluth, Martin Lindberg-Larsen

**Affiliations:** 1 Orthopaedic research unit, Department of Orthopaedic Surgery and Traumatology, Odense University Hospital, Odense, Denmark; 2 Department of Clinical Research, University of Southern Denmark, Odense, Denmark

**Keywords:** Resection knee arthroplasty, Distal femoral replacement, Revision knee arthroplasty, Periprosthetic fracture, Patient safety

## Abstract

**Background:**

Distal femoral resection knee arthroplasty may be a viable option for several indications other than bone tumors. Resection knee arthroplasty appears to be becoming more common, but patients requiring this type of surgery are often elderly and with high comorbidity. The aim of this study was to report in-hospital complications, readmissions, reoperations, and mortality after distal femoral resection knee arthroplasty for non-tumor indications.

**Methods:**

We retrospectively identified a consecutive cohort of 45 knees (45 patients) treated with distal femoral resection knee arthroplasty in a single institution between 2012 and 2021. Indications for surgery were failure of osteosynthesis (8), primary fracture treatment (2), periprosthetic fracture (22), and revision arthroplasty with severe bone loss (13). A major reoperation was defined as a major component exchange procedure or amputation. Mean follow-up was 3.9 years.

**Results:**

The mean age was 71.3 years (SD 12.3), and 64.4% were female; 8.9% were ASA I, 40% ASA II, and 51% ASA III. Median length of stay was 7 days (range 3–19) with no major in-hospital complications, but 55.6% (*n* = 25) required blood transfusion. The 90-day readmission rate was 17.8% (*n* = 8), of which 50% was prosthesis-related. Four patients (8.9%) underwent major reoperation due to infection (*n* = 2), mechanical failure (*n* = 1), or periprosthetic fracture (*n* = 1). The mortality rate was 0% ≤ 90 days and 2.2% ≤1 year.

**Conclusions:**

Distal femoral resection knee arthroplasty in this fragile patient population appears to be a viable and safe option considering that it is a limp salvage procedure most cases.

## Introduction

Distal femoral resection knee arthroplasty may be a viable option for several non-oncologic indications such as comminuted distal femoral fractures where sufficient osteosynthesis is not possible, periprosthetic fractures around the femoral component of a total knee arthroplasty, and revision knee arthroplasties with severe bone loss in the distal femur.

The number of resection knee arthroplasties performed due to distal femoral fractures and periprosthetic distal femoral fractures appears to be increasing [1, 2]. As the number of revision knee arthroplasties is projected to increase dramatically within the next decades, the number of revision cases with severe bone loss will also increase [3].

The patients requiring this type of surgery are often elderly and with high medical comorbidity. Common fixation strategies that prohibit early ambulation may compromise clinical outcomes, and high reoperation rates have been reported when using locking plates on comminuted fractures [3]. Similar to geriatric hip fractures, the risk of perioperative complications and mortality after distal femoral fractures is high [4]. Only limited data exist on the use of femoral resection knee arthroplasty for non-oncologic indications, but most recently published case series (*n* = 11–54) suggest it may be a reasonable treatment option [5–10].

The aim of the current study was to report in-hospital complications, readmissions, reoperations, and mortality after distal femoral resection knee arthroplasty for non-tumor indications in a Danish setting.

## Patients and methods

### Study design

The design was a retrospective, single-center study on a consecutive cohort of patients treated with distal femoral resection knee arthroplasty between January 2012 and October 2020.

### Patients and surgical procedures

**Fig. 1:**
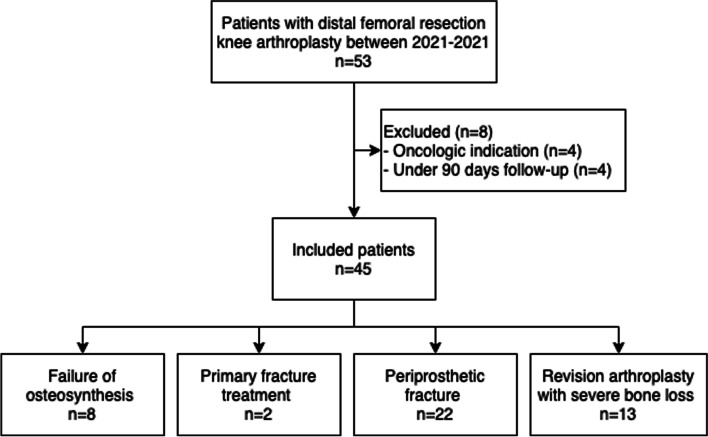
Flowchart of patient inclusion in the study and indications for distal femoral resection knee arthroplasties

A total of 53 distal femoral resection knee arthroplasties were performed during the study period. After exclusion of three patients treated for oncologic indications, 45 distal femoral resection knee arthroplasties performed in 45 patients were available for analysis. The surgical indications were failure of osteosynthesis (*n* = 8), primary fracture treatment (*n* = 2), periprosthetic fracture (*n* = 22), and revision arthroplasty with severe bone loss (*n* = 13) (Fig. 1). All procedures were performed in a tertiary referral center by two consultant knee revision surgeons (CE and MLL). The GMRS – Global Modular Replacement System (Stryker) prosthesis was used in the first 14 cases and the LPS – Limp Preservation System (DePuy Synthes) in the last 31 cases. All cases were performed without the use of a tourniquet and 1 g tranexamic acid were administered preoperatively. Patients received prophylactic antibiotic treatment with 1 g Dicloxacillin (1.5 g Cerfuroxime in case of allergy) preoperatively and at 8, 16, and 24 h after surgery in non-revision cases. In the revision cases (prosthesis exchange procedures), prophylactic antibiotic treatment were continued until analysis of intraoperative biopsies (*n* = 5) were finalized and microbiology results were confirmed as negative.

Thromboprophylaxis was given as dalteparin (Fragmin, Pfizer Health Care, New York, USA) 5000 IU/day for 7 days after surgery. Five tissue biopsies were obtained during surgery in all cases, and patients were treated with oral antibiotics until the microbiology results were confirmed as negative. All included patients received physiotherapy within 24 h of surgery to help standing up and walk using a walking aid. Full weight bearing on the operated leg was allowed and encouraged immediately after surgery. A rehabilitation plan was performed during admission and rehabilitation was continued in the municipality. Patients were followed-up with a clinical evaluation at 3 months and an x-ray and clinical evaluation 1 years after surgery.

In the 24 acute cases (primary fractures and periprosthetic fractures), the median time from diagnosis (x-ray examination) to surgery was 108 h (44–696 h). Both x-ray and CT-scan was performed preoperatively in all cases.

### Outcomes

Patient records were scrutinized for preoperative patient characteristics as well as peri- and postoperative outcomes including in-hospital complications, readmissions, and referrals to other hospitals. Postoperative length of hospital stay was defined as the total number of postoperative nights in hospital including referrals to other hospitals. All unplanned readmissions within 90 days of surgery were registered. All reoperations within the follow-up period of 3.9 years (206 days – 9.9 years) were registered and classified as either major or minor reoperations. A major reoperation was defined as a major component (tibial or femoral component) exchange procedure or a femoral amputation. Postoperative mortality rates within 90 days and within 1 year were analyzed.

#### Statistics

Continuous data are presented as mean (SD) or median (interquartile range (IQR)) as appropriate. Categorical data are presented as n (%) with 95% confidence intervals (CI).

Data were analyzed using SPSS version 24 (2016; Armonk, NY: IBM Corp.).

## Results

The data comprised 44 distal femoral resection knee arthroplasties performed in 44 patients with a mean age of 71.3 (SD 12.3) (Table 1).

**Table 1:** Characteristics of patients undergoing distal femoral resection knee arthroplasty for non-tumor indications

	All indications	Osteosyntheses failure	Fracture	Periprosthetic fracture	Revision arthroplasty
n (%)	45 (100)	8 (17.8)	2 (4.4)	22 (48.9)	13 (28.9)
Mean age (years) (SD)	71.3 (12.3)	61.8 (18.5)	74.5 (6.4)	74.8 (9.4)	70.9 (10.8)
Female (%)	29 (64.4)	7 (87.5)	1 (50)	15 (68.2)	6 (46.2)
Median BMI (Range)	27 (18–46)	24.0 (20–30)	25.5 (22–29)	27.0 (21–33)	27.0 (18–46)
ASA (%)					
ASA Score 1	4 (8.9)	2 (25)	0	0	2 (15.4)
ASA Score 2	18 (40)	4 (50)	1 (50)	9 (40.9)	4 (30.8)
ASA Score 3	23 (51)	2 (25)	1 (50)	13 (59.1)	7 (53.8)
ASA Score 4	0	0	0	0	0
Insulin-dependent diabetes (%)	1 (2.2)	0	0	1 (4.5)	0
Non-insulin dependent diabetes	2 (4.4)	0	0	1 (4.5)	1 (7.7)
Cardiac disease (%)	15 (33.3)	3 (37.5)	0	9 (40.9)	3 (23.1)
Pulmonary disease (%)	7 (15.6)	2 (25)	1 (50)	4 (18.2)	0
Immunosuppression (%)	3 (6.7)	1 (12.5)	0	1 (4.5)	1 (7.7)
Preoperative hemoglobin level, g/dl (SD)	7.4 (1.1)	8.1 (0.9)	9.1 (1.4)	6.8 (1.1)	7.6 (0.9)

*BMI* Body mass index, *ASA* American Society of Anesthesiologists

Median length of stay was 7 days (range 3–19). During primary admission, one patient had a patella dislocation and underwent a minor reoperation with lateral capsular release, medial capsular duplication, and liner exchange. No major in-hospital complications were registered. A total of 55.6% (*n* = 25) required blood transfusion postoperatively (Table 2).

**Table 2:** Outcomes and surgical characteristics for patients according to indication for surgery

	All indications	Osteosyntheses failure	Fracture	Periprosthetic fracture	Revision arthroplasty
n (%)	45 (100)	8 (17.8)	2 (4.4)	22 (48.9)	13 (28.9)
Median postop. LOS, days (range)	7 (3–19)	6 (3–8)	6 (5–7)	7 (3–15)	5 (3–19)
Mean postop. LOS, days (SD)	6.8 (3.5)	5.5 (1.9)	6 (1.4)	7.7 (3.7)	6.2 (4.1)
Readmission ≤90 days, n (%)	8 (17.8)	2 (25)^a^	1 (50)^b^	2 (9.1)^c^	3 (23.1)^d^
Mortality ≤90 days, n (%)	0	0	0	0	0
Mortality ≤1 year, n (%)	1 (2.2)	0	0	1 (4.5)	0
All-cause reoperation rate (%)	8 (17.8)	4 (50)	0	3 (13.6)	1 (7.7)
Reoperation, major revisions (%)	4 (8.9)	3 (37.5)	0	1 (5)	0
Mean duration of surgery, minutes (SD)	176.8 (43.4)	157 (42.0)	189 (72.1)	165.8 (28.9)	197.9 (53)
Median number blood transfusion, units (range)	1 (0–9)	1 (0–6)	1 (0–2)	1 (0–9)	1 (0–6)
Postoperative hemoglobin level, g/dl (SD)	5.3 (1.09)	5.3 (0.9)	5.2 (1.6)	5.2 (0.4)	5.5 (1.5)

^a^2 cases of prosthetic infection

^b^1 case due to mobilization problems

^c^1 case of prosthesis complications; 1 case of patella dislocation

^d^1 case of prosthetic infection; 1 case of sepsis; 1 case of gastrointestinal problems Postop. LOS = postoperative length of hospital stay

The 90-day readmission rate was 17.8% (*n* = 8). Half of the complications causing readmission were related to the prosthesis (infection or liner breakage/implant failure requiring liner exchange and synovectomy) (Table 2).

The all-cause reoperation rate was 17.8% (*n* = 8), but only 8.9% (*n* = 4) were major reoperations. Causes of reoperations are presented in Table 3.

**Table 3:** Reasons for reoperation for patients undergoing distal femoral resection knee arthroplasty, according to surgical indication

Indication for index surgery	Reoperation rate (%)	First reoperation	Second reoperation
Osteosyntheses failure (*n* = 8)	50%	1: 19 days after index surgery, minor reoperation due to infection	
		2: 1071 days after index surgery, major reoperation with prothesis exchange due to loosening of prothesis	
		3: 15 days after index surgery, major reoperation with prothesis removal due to infection	23 days after index surgery and 8 days after first reoperation, amputation due to infection
		4: 889 days after index surgery, major reoperation with prosthesis removal due to infection (first stage of two-stage procedure)	974 days after index operation, second stage of two-stage procedure
Fracture (*n* = 2)	0	0	
Periprosthetic fracture (*n* = 22)	13.6%	1: 29 days from index surgery, reoperation with total femur prothesis after fall and fracture of above-knee prosthesis and under-hip implant	33 days from index surgery, minor reoperation due to cicatrice rupture
		2: 11 days from index surgery, prothesis exchange due to prothesis dislocation	
		3: 14 days from index surgery, minor reoperation due to patella dislocation	
Revision arthroplasty (*n* = 13)	7.7%	1: 38 days from index surgery, minor reoperation due to infection	

The 90-day mortality rate was 0%, and the 1-year mortality rate was 2.2%.

## Discussion

This study found that distal femoral resection knee arthroplasty appeared to have acceptable patient safety as it was associated with no major in-hospital complications, 17.8% readmission rate in the first 90 days, 8.9% major reoperations, and 2.2% mortality in the first year after surgery. These results are comparable with the results reported from similar studies [2, 11, 12] (Table 4).

**Table 4:** Comparison of current study results with previous studies on femoral resection knee arthroplasty for non-oncologic indications

	No. and mean patient age	ASA grade, %	Median postop. LOS	In-hospital complications	Blood trans.	Readmission rate ≤ 90 days	All-cause reop. Rate	Mortality ≤ 90 days	Mortality ≤ 1 year
**Current study**	*N* = 45 71.3 y	I:8.9 II:40 III:51 IV:0	7 days	2% (1)	56%	18%	18%	0	2%
**Darrith et al. 2020** **[**10**]**	*N* = 22 75.8 y	–	6 days	–	–	–	14%	–	–
**Angers- Goulet et al. 2019** **[**2**]**	*N* = 19 79.7 y	–	–	–	–	–	32%	–	–
**Chalmers et al. 2020** **[**11**]**	*N* = 34 79.5 y	I: 0 II:26 III:71 IV:9	8 days	12%	51%	–	7%	0	2%
**Hoellwarth et al. 2018** **[**8**]**	*N* = 53 80.1 y	–	6 days	–	28%	11%	6%	4%	10%
**Rajasekaran et al. 2020** **[**12**]**	*N* = 24 71.8 y	I:0 II: 54.1 III:45.8 IV:0	10 days	29%	–	–	8%	0%	0%

*ASA* American Society of Anesthesiologists, *postop. LOS* Postoperative length of hospital stay, *Blood trans* Blood transfusion, *reop* reoperation

The postoperative length of hospital stay of 6.5 days in our study was similar to [8, 10] or shorter [11, 12] than previously reported (Table 4). When considering the fragile patient group and the major surgical trauma, a 6.5-day length of stay is reasonable and, as expected, is longer than the 4 days reported after revision total knee arthroplasty on a nationwide basis in Denmark [13].

Chalmers et al. [11] reported postoperative medical complications such as pulmonary embolism, cerebrovascular accidents, and acute renal failure as causes of prolonged length of stay, but this was not the case in our study with no serious postoperative medical complications. Our finding that 55% of patients required blood transfusions seems high, but at the same level as previously reported (28 and 51%) [8, 11]. A tourniquet was not used in any of the cases in our series, in contrast to previous studies [2], and data from our study cannot recommend for or against the use of a tourniquet in these procedures.

The rate of readmission within 90 days was 17.8% in our study compared to 11% reported in the only other study reporting rate of readmission [8]. For comparison, the rate of readmission within 90 days has been reported to be 10% after revision knee arthroplasty [13] and 18% after revision hip arthroplasty [14] nationwide in Denmark, while that after geriatric hip fractures has been reported as 24.1% [15]. Therefore, a readmission rate of 17.8% may be acceptable. Surgical site infections caused readmission in 3 (6.7%) cases, however, and this is a serious condition resulting in prolonged morbidity for the patients.

The all-cause reoperation rate was 17.8% (*n* = 8), and 8.9% (*n* = 4) had major reoperations. Reoperation rates in other recent studies on distal femoral resection knee arthroplasty range from 5.6 to 31.6% [2, 8, 10–12]. The most frequent complication causing reoperation in our study was infection (8.9%), which is in accordance with previous findings [10, 11], and may be explained by the extensive surgical trauma, long operating time, and large amount of foreign body implanted.

Hoellwarth et al. [8] considered periprosthetic distal femoral fractures treated with distal femoral resection knee arthroplasties to be a useful treatment option, but found that about 20% died within a year of injury, about 10% needed further surgery, and about 30% did not regain their former mobility (30%). In our study, we found 0% mortality at 90 days and 2.2% at 1 year, which are both low compared to the fragile hip fracture group [16] and other studies presenting results of distal femoral resection knee arthroplasty [8, 11, 12].

The limitations of our study include the retrospective design, small cohort, and single-center approach involving two surgeons, and these may limit the generalizability of our results to other settings. Distal femoral resection knee arthroplasty is a highly specialized treatment option, however, and should be performed in specialist referral centers. The strengths of our study are the consecutive cohort and the detailed information on patient characteristics, comorbidity, readmissions, and reoperations. We chose to include patients with short follow-up (minimum follow-up 206 days) less than 1 year as this procedure is an acute procedure in most cases and most complications occur early. Hence, if we had excluded patients with less than 1 year of follow-up we would have underestimated the re-operation rate. Future studies should address the patient perspective, for example by incorporating measures of patient outcome, and prospective clinical trials should investigate whether the indications for this procedure could be expanded when comparing with alternative surgical options.

## Conclusion

Distal femoral resection knee arthroplasty is a treatment option that allows early mobilization in a fragile group of patients. It appears to be a viable and safe option considering that it is a limp salvage procedure most cases.

## Data Availability

The data used and/or analyzed during the current study is available from the corresponding author (Yasemin Corap, Yasemin.Corap3@rsyd.dk) on reasonable request.
